# Fluctuations in the detection of the HOM effect

**DOI:** 10.1038/s41598-020-77189-6

**Published:** 2020-11-18

**Authors:** Dmitry N. Makarov

**Affiliations:** Northern (Arctic) Federal University, Arkhangelsk, 163002 Russia

**Keywords:** Quantum optics, Single photons and quantum effects, Optical physics

## Abstract

Hong-Ou-Mandel (HOM) effect is known to be one of the main phenomena in quantum optics. It is believed that the effect occurs when two identical single-photon waves enter a 1:1 beam splitter, one in each input port. When the photons are identical, they will extinguish each other. In this work, it is shown that these fundamental provisions of the HOM interference may not always be fulfilled. One of the main elements of the HOM interferometer is the beam splitter, which has its own coefficients of reflection $R = 1/2$ and transmission $T = 1/2$. Here we consider the general mechanism of the interaction of two photons in a beam splitter, which shows that in the HOM theory of the effect it is necessary to know (including when planning the experiment) not only $R = 1/2$ and $T = 1/2$, but also their root-mean-square fluctuations $\Delta R ^ 2, \Delta T ^ 2$, which arise due to the dependence of $R = R(\omega _1, \omega _2)$ and $T = T (\omega _1, \omega _2)$ on the frequencies where $\omega _1, \omega _2$ are the frequencies of the first and second photons, respectively. Under certain conditions, specifically when the dependence of the fluctuations $\Delta R^2$ and $\Delta T^2$ can be neglected and $R=T=1/2$ is chosen, the developed theory coincides with previously known results.

## Introduction

The HOM effect was first experimentally demonstrated by Hong et al in 1987^1^. HOM interference shows up in many instances, both in fundamental studies of quantum mechanics and in practical implementations of quantum technologies^2–8^. For example, one of the main practical applications of the HOM effect is to check the degree of indistinguishability of two incoming photons. When the HOM dip reaches all the way down to zero coincident counts, the incoming photons are perfectly indistinguishable, whereas if there is no dip, the photons are distinguishable. A HOM interferometer scheme was presented in^1^, one of the main elements of which was a beam splitter (BS). To observe quantum interference, a BS is chosen close to 1:1 (having coefficients of reflection *R* and transmission *T* close to 1/2). A theoretical explanation of the HOM effect based on constant coefficients *R* and *T* and boson statistics of photons is quite simple^9,10^. In this interpretation, we are not interested in what happens to the incident photons in the BS. For this, they consider BS lossless (hereinafter simply BS) as ideal, i.e. with constant coefficients *R* and *T* and BS is the source of the other two photons obeying bosonic statistics. In this case, the annihilation operators before entering 1 and 2 photons in BS represent ${\hat{a}}_1$ and ${\hat{a}}_2$, respectively, and after exiting BS is ${\hat{b}}_1$ and ${\hat{b}}_2$. The transformation from one pair of operators to another is generally described by the BS matrix (denoted as $U_{BS}$) in the form (see, e.g.^11,12^)

1 $$\begin{aligned} \begin{pmatrix} {\hat{b}}_1\\ {\hat{b}}_2 \end{pmatrix}= U_{BS} \begin{pmatrix} {\hat{a}}_1\\ {\hat{a}}_2 \end{pmatrix}, ~~~ U_{BS}= \begin{pmatrix} e^{i\phi _1}\sqrt{T}&{} e^{i\phi _2}\sqrt{R}\\ - e^{-i\phi _2}\sqrt{R}&{} e^{-i\phi _1}\sqrt{T} \end{pmatrix} . \end{aligned}$$

**Figure 1:**
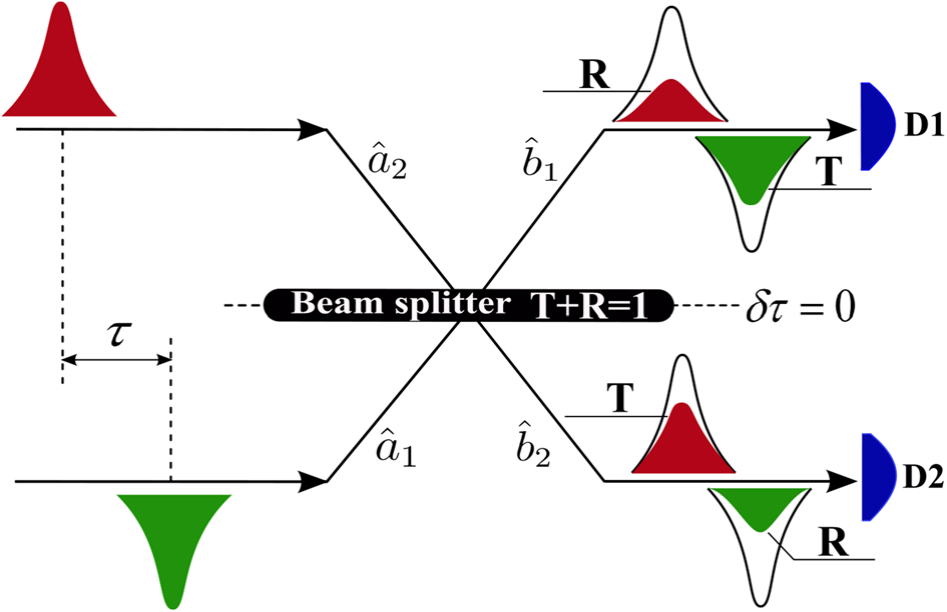
Schematic representation of the HOM interferometer, where $D_1, D_2$ are the first and second detectors, respectively; $\tau$ is the time delay between 1 and 2 photons and $\delta \tau$ is the time delay caused by the spatial displacement of the BS from the equilibrium position.

It is easy to see that for $R=T=1/2$, the photons at the output (described by the operator ${\hat{b}}_2{\hat{b}}_1$) only come out in pairs from 1 or 2 ports. This analysis is fundamental to understanding the HOM effect and is not subject to any additional research. The basic scheme HOM interferometer for arbitrary photons (including quantum entangled photons) is shown in Fig. 1. In reality, the pair of photons arriving at the BS do not have a set frequency, but have a certain frequency distribution. Nonetheless, in the theoretical description (e.g.^1,13–17^) of the experimentally observed value *P* (*P* is the joint probability of detecting photons after exiting the BS on the output ports), the frequency distribution does not affect BS matrix $U_{BS}$, because *R* and *T* are constant values. Currently, the well-known HOM effect theories are based on calculating the value of *P* within the constant values of $R=T=1/2$.

In the work presented the coefficients *R* and *T* are variables, which significantly affects the theory of the HOM effect. The problem of interaction of two photons in BS is solved analytically, allowing the determination of the photon statistics after exiting the BS. Within the general form $U_{BS}$ is a BS matrix similar to Eq. (1), where *R* and *T* are some functions that depend on the frequencies of incident photons, the interaction time of two photons in BS, and on the BS material. This leads to the value of the coincidence counting probability *P* being calculated to take into account the dependence on the frequencies of *R* and *T*. It is shown that even in the case of identical incident photons and their average values ${\bar{R}}={\bar{T}}=1/2$ (averaging over the frequencies of incident photons), a zero value of *P* may not be observed, despite being predicted by the HOM interference theory taking into account the constants *R* and *T*. Indeed, in the case of constant coefficients, as well as without a time delay between two photons, i.e. $\delta \tau = 0$ or $\tau = 0$ and identical photons, because it is well known that $P \propto (R-T)^2$, for $R=T=1/2$ we get $P=0$^1,13^. In our case, $P \propto \overline{(R-T)^2}$, which means $P \propto \overline{R^2}-({\overline{R}})^2$ or $P \propto \overline{T^2}-({\overline{T}})^2$ (when ${\bar{R}}={\bar{T}}=1/2$) i.e. there is a fluctuation in the reflection and transmission coefficients, had was not earlier taken into account in theoretical and experimental studies. It is shown (arbitrary falling photons, including not only Fock state, but also taking into account the time delay $\delta \tau$ and $\tau$) that under certain conditions the coefficients *R* and *T* can be considered constant, and the results obtained pass into well-known approaches. The theory developed here is especially important when planning experiments in the HOM interferometer and analyzing them; because the fluctuations of *R* and *T* can be very large, the results of such experiments may not be correctly interpreted.

## Photons in BS

It is well known that in quantum optics two modes of the electromagnetic field (two input ports) are usually considered, since even if 1 port remains unused, it should be considered as an input for vacuum fluctuations^9,10^. Thus, we will proceed from the fact that we have two ports at the input. Two input and output ports can be in the form of freely spreading photons or in the form of waveguides along which photons propagate. If the waveguides are connected, then we get BS in the form of a coupled waveguide. It should be added that such systems are usually studied using various simplified models, for example, Bose–Hubbar model^18^, Jaynes–Cummings model (JCM)^19^, Dicke model^20^ and others. We will try to approach this problem based on a complete record of the Schrödinger equation of all interacting particles in the system under consideration.

Consider a polyatomic system (e.g. BS) interacting with two photons. We represent the electromagnetic field of photons through the transverse vector potential $\mathbf{A }$ in the Coulomb gauge $div \mathbf{A } = 0$^9,10^, then the Hamiltonian of such a system will be (further, the atomic system of units to be used will be: $\hbar$ = 1; |*e*| = 1; $m_e$ = 1, where $\hbar$ is Dirac’s constant, *e* is the electron charge, $m_e$ is the electron mass)

2 $$\begin{aligned} \left\{ {{\hat{H}}}_{1}+{{\hat{H}}}_{2}+\frac{1}{2}\sum _a\left( {\hat{\mathbf{p }}}_a+\frac{1}{c}\hat{\mathbf{A }}_a\right) ^2 + \sum _a U(\mathbf{r }_a)\right\} \Psi = i\frac{\partial \Psi }{\partial t}, \end{aligned}$$

where ${{\hat{H}}}_{i} =\omega _i\hat{a_i}^{+}\hat{a_i}$ is the Hamilton operator for the first ($i=1$) and the second ($i = 2$) photon ($\omega _i$ is the frequency, and $\hat{a_i}$ is the annihilation operator of the photon with number *i*); $U (\mathbf{r }_a)$ is the atomic potential acting on the electron with number *a* ; ${\hat{\mathbf{p }}}_a$ is the electron momentum operator with the number *a*; $\hat{\mathbf{A }}_a = \hat{\mathbf{A }}_{1, a} + \hat{\mathbf{A }}_{2,a}$, where $\hat{\mathbf{A }}_{i,a} = \sqrt{\frac{2 \pi c^2}{\omega _i V_i}}\mathbf{u }_i(\hat{a_i}^{+}+\hat{a_i})$ is the vector potential in the dipole approximation created by the *i* photon acting on the electron with number *a* (*c* is the speed of light, $V_i$ is the modal volume, $\mathbf{u }_i$ is the polarization of the photon with the number *i*)^9,10^; and the sum $\sum _a$ (Eq. 2) is over all electrons polyatomic system. It should be added that the dipole approximation gives correct results at photon wavelengths $\lambda \gg 1$ i.e. much larger than atomic sizes. Furthermore Eq. (2) is more convenient to consider in the form of a differential equation (which was the approach taken in^21–23^), going from the operators ${{\hat{a}}} = \frac{1}{\sqrt{2}}(q+\frac{\partial }{\partial q}), {{\hat{a}}}^{+} = \frac{1}{\sqrt{2}} (q-\frac{\partial }{\partial q})$ to the electromagnetic field variables *q*^9,10^. As a result, the Hamiltonian of Eq. (2) will be

3 $$\begin{aligned} {{\hat{H}}}= \sum ^{2}_{i=1}\left\{ \frac{\omega _i}{2}\left( q^2_i-\frac{\partial ^2}{\partial q^2_i} \right) +{\overline{N}}\frac{\beta ^2_i}{2}q^2_i+\beta _i q_i\mathbf{u }_i\sum _a {\hat{\mathbf{p }}_a}\right\} + {\overline{N}}\beta _1 \beta _2 q_1 q_2 \mathbf{u }_1 \mathbf{u }_2+\sum _a\frac{{\hat{\mathbf{p }}}^2_a}{2}+\sum _a U(\mathbf{r }_a), \end{aligned}$$

where $\beta _i = \sqrt{\frac{4 \pi }{\omega _i V_i}}$, and the value ${\overline{N}}= \sum _a (1)$ is the number of electrons participating in the interaction with photons in a polyatomic system. Eq. (3) can be seen to correspond to the equation for coupled harmonic oscillators interacting with the electrons of a polyatomic system. A similar system was considered in^24^, but without taking into account interaction with electrons, we obtain

4 $$\begin{aligned} {{\hat{H}}}= \sum ^{2}_{i=1} \frac{1}{2}\left\{ \sqrt{A_i} \left( {{\hat{P}}}^2_i+y^2_i\right) +2 \mathbf{D }_i y_i\sum _a {\hat{\mathbf{p }}_a}\right\} + \sum _a \frac{{\hat{\mathbf{p }}}^2_a}{2}+\sum _a U(\mathbf{r }_a) , \end{aligned}$$

where $y_1 = A^{1/4}_1\left( q_1/\sqrt{\omega _1} \cos \alpha -q_2/\sqrt{\omega _2} \sin \alpha \right)$ and $y_2 = A^{1/4}_2\left( q_1/\sqrt{\omega _1} \sin \alpha + q_2/\sqrt{\omega _2} \cos \alpha \right)$ are new variables; ${{\hat{P}}}_i=-i\partial /\partial y_i$; $\mathbf{D }_1=\beta _1 A^{-1/4}_1\sqrt{\omega _1}\mathbf{u }_1\cos \alpha - \beta _2 A^{-1/4}_1\sqrt{\omega _2}\mathbf{u }_2 \sin \alpha$; $\mathbf{D }_2=\beta _1 A^{-1/4}_2\sqrt{\omega _1}\mathbf{u }_1\sin \alpha +\beta _2A^{-1/4}_2 \sqrt{\omega _2}\mathbf{u }_2 \cos \alpha$. The study^24^ showed that $\tan (2\alpha )=C/(B_2-B_1)$, where $C=2{\overline{N}} \beta _1 \beta _2 \sqrt{\omega _1 \omega _2} \mathbf{u }_1 \mathbf{u }_2$; $B_i=(\omega _i+{\overline{N}}\beta ^2_i)\omega _i$ and $A_i=B_i+(-1)^i C/2 \tan \alpha$. Obviously, the value of $\beta _i$ is very small in the case of single-photon interaction, see, e.g.^25^, where $\beta \ll 1$, even in the case of strong focusing. In this case, the quantities $\mathbf{D }_1$ and $\mathbf{D }_2$ are negligible. This is an obvious fact, since these quantities are responsible for various inelastic transitions of electrons in an atom under the action of photons, which are usually negligible in lossless BS. As a result, the dynamics of two photons in BS will be described by the wave function

5 $$\begin{aligned} |\Phi (t_{BS})\rangle =e^{-i {{\hat{H}}_{BS}}t_{BS}}|\Phi (0)\rangle ; ~~~ {\hat{H}}_{BS} = \sum ^{2}_{i=1} \frac{\sqrt{A_i}}{2}\left\{ {{\hat{P}}}^2_i+y^2_i\right\} , \end{aligned}$$

where $$ t_{BS}
$$ is the photon interaction time in BS and $$ |\Phi (0)\rangle $$ is the initial state of the photons before entering the BS. In the future, to calculate the required quantities, we will need the electric field operators $$ {{\hat{E}}}^{+}_1(t_1) $$ and $$ {{\hat{E}}}^{+}_2(t_2) $$ at time instants $$ t_1 $$ and $$ t_2 $$ on the first and second detectors, respectively. To do this, we need to find the evolution (in BS, as well as from BS to detectors) of the operators $$ {{\hat{E}}}^{+}_{01}(0) $$ and $${{\hat{E}}}^{+}_{02}(0) $$ of the first and second photons, respectively

6 $$\begin{aligned} {{\hat{E}}}^{+}_1(t_1)=e^{i {{\hat{H}}_0}t_1}e^{i {{\hat{H}}_{BS}}t_{BS}}{{\hat{E}}}^{+}_{01}(0)e^{-i {{\hat{H}}_{BS}}t_{BS}} e^{-i {{\hat{H}}_0}t_1};~~~ {{\hat{E}}}^{+}_2(t_2)=e^{i {{\hat{H}}_0}t_2}e^{i {{\hat{H}}_{BS}}t_{BS}}{{\hat{E}}}^{+}_{02}(0)e^{-i {{\hat{H}}_{BS}}t_{BS}} e^{-i {{\hat{H}}_0}t_2}, \end{aligned}$$

where ${{\hat{H}}_0} = \sum ^{2}_{i = 1} \omega _i/2\left\{ - \partial ^2/ \partial q^2_i + q^2_i \right\}$ is the Hamiltonian of photons outside of BS. Because ${{\hat{E}}}^{+}_{01}(0) \propto {{\hat{a}}}_1$, and ${{\hat{E}}}^{+}_{02}(0) \propto {{\hat{a}}}_2$ (see, e.g.^9,10^), it is more convenient to consider not the electric field operators, but the photon creation and annihilation operators before entering BS (${{\hat{a}}}_1$ and ${{\hat{a}}}_2$) and on the detectors (${{\hat{b}}}_1$ and ${{\hat{b}}}_2$). To this end, we replace ${{\hat{E}}}^{+}_{01}(0)\rightarrow {{\hat{a}}}_1, {{\hat{E}}}^{+}_{02}(0)\rightarrow {{\hat{a}}}_2$ and ${{\hat{E}}}^{+}_{1} (t_1) \rightarrow {{\hat{b}}}_1(t_1), {{\hat{E}}}^{+}_{2}(t_2) \rightarrow {{\hat{b}}}_2(t_2)$. Taking into account the time delay $\delta \tau$ for the spatial displacement of BS from the equilibrium position and the time delay $\tau$ between 1 and 2 photons (see Fig. 1)

7 $$\begin{aligned} {{\hat{b}}}_1(t_1)= & {} e^{i\phi _1}\sqrt{T} e^{-i\omega _1 (t_1-\tau )}{{\hat{a}}}_1+e^{i\phi _2}\sqrt{R} e^{-i\omega _2 (t_1+\delta \tau /2)}{{\hat{a}}}_2, \nonumber \\ {{\hat{b}}}_2(t_2)= & {} e^{-i\phi _1}\sqrt{T} e^{-i\omega _2 t_2}{{\hat{a}}}_2 -e^{-i\phi _2}\sqrt{R} e^{-i\omega _1 (t_2-\delta \tau /2-\tau )}{{\hat{a}}}_1, \end{aligned}$$

where $\phi _1, \phi _2$ are some non-essential phases, and the coefficients

8 $$\begin{aligned} R=\frac{\sin ^2\left( \Omega t_{BS}/2 \sqrt{1+\epsilon ^2}/2 \right) }{(1+\epsilon ^2)};~\Omega =\frac{8\pi {\overline{N}} \mathbf{u }_1 \mathbf{u }_2 }{(\omega _1+\omega _2)\sqrt{V_1 V_2}};~T=1-R;~ \epsilon =\frac{\omega _2 -\omega _1}{\Omega }+\frac{\sqrt{V_2/V_1}-\sqrt{V_1/V_2}}{2 \mathbf{u }_1 \mathbf{u }_2 } . \end{aligned}$$

From Eq. (7) it can be seen that the matrix BS that is $U_ {BS}$ completely corresponds to the matrix (Eq. 1) (needless to say, for $t_1 = t_2 = t$ and for $\delta \tau = \tau = 0$). In addition, *T* and *R* are symmetric, i.e. if we change the first to the second photon $\omega _1 \rightarrow \omega _2, V_1 \rightarrow V_2, \mathbf{u }_1 \rightarrow \mathbf{u } _2$ and vice versa, then, as anticipated, the coefficients will not alter.

## Two-photon interference

The next problem, we consider is the probability $P_ {1,2}$ of the joint detection of photons on 1 and 2 detectors (correlation between the two detectors). If our coincidence gate window accepts counts for a time $T_D$, then the rate of coincidences P, between detectors 1 and 2 is proportional to (see, e.g.^1,13,14^)

9 $$\begin{aligned} P_{1,2}\propto \int ^{T_{D}/2}_{-T_{D}/2}\int ^{T_{D}/2}_{-T_{D}/2}\langle {{\hat{b}}}^{\dagger }_1(t_1){{\hat{b}}}^{\dagger }_2(t_2){{\hat{b}}}_1(t_1){{\hat{b}}}_2(t_2) \rangle dt_1 dt_2. \end{aligned}$$

Let us consider the case where the reaction time $\tau _D$ (time resolution) of the detectors $D_1$ and $D_2$ in the experiment is many times slower than other time scales of the problem $\tau _D \gg 1$: in this case $T_D \rightarrow \infty$. It should be added that the theory presented below is not difficult to generalize to the case of $\tau _D \ll 1$, which is currently implemented experimentally (e.g.^16,26^).

Equation (9) is applicable in the case of monochromatic photons. In reality, they cannot be such and it is necessary to take into account the frequency distribution, and in this case the initial wave function of the photons will be in the form $| \Psi \rangle = \int \phi (\omega _1, \omega _2) {{\hat{a}}}^{\dagger }_2{{\hat{a}}}^{\dagger }_1 | 0 \rangle d \omega _1 d \omega _2$, where $\phi (\omega _1,\omega _2)$ is the joint spectral amplitude (JSA) of the two-photon wavefunction ($\int | \phi (\omega _1, \omega _2) |^2 d \omega _1 d \omega _2 = 1$). Further calculations of $P_{1,2}$ are similar to those that are generally accepted (e.g.^13,14^), the only difference being that it is necessary to consider the *T* and *R* functions depending on the frequencies. As a result, we obtain

10 $$\begin{aligned} P_{1,2}= & {} \int ^{\infty }_{-\infty }\int ^{\infty }_{-\infty } \left| \xi _1(t_1,t_2,\tau )-\xi _2(t_1,t_2,\tau ,\delta \tau ) \right| ^2 dt_1 dt_2~, \nonumber \\ \xi _1(t_1,t_2,\tau )= & {} \frac{1}{2\pi }\int \phi (\omega _1,\omega _2)T(\omega _1,\omega _2)e^{-i\omega _1(t_1-\tau )}e^{-i\omega _2 t_2} d\omega _1 d \omega _2~, \nonumber \\ \xi _2(t_1,t_2,\tau ,\delta \tau )= & {} \frac{1}{2\pi }\int \phi (\omega _1,\omega _2) R(\omega _1,\omega _2) e^{-i\omega _2(t_1+\delta \tau )}e^{-i\omega _1(t_2-\delta \tau -\tau )} d\omega _1 d \omega _2, \end{aligned}$$

where $P_{1,2}$ is normalized so that with $t_{BS} = 0$ the probability is $P_{1,2} = 1$ (without BS, the probability of joint operation of the detectors is $100 \%$), which corresponds to standard normalization in HOM theory. We then obtain

11 $$\begin{aligned} P_{1,2}= & {} \int \Biggr ( |\phi (\omega _1,\omega _2)|^2 \left( T^2(\omega _1,\omega _2)+R^2(\omega _1,\omega _2) \right) - \nonumber \\&\quad 2\mathrm{Re}\biggr \lbrace \phi (\omega _1,\omega _2) \phi ^*(\omega _2,\omega _1) T(\omega _1,\omega _2)R(\omega _2,\omega _1) e^{-i(\omega _2-\omega _1)(\delta \tau +\tau )}\biggl \rbrace \Biggl ) d \omega _1 d \omega _2 . \end{aligned}$$

It should be added that if *T* and *R* are assumed to be independent of frequencies and $T = R = 1/2$, then Eq. (11) corresponds to the well-known equation, e.g.^27–29^. It is also seen that the time delay of $\delta \tau$ and $\tau$ is additively $\delta \tau + \tau$; therefore, we denote it by $\Delta \tau = \delta \tau + \tau$.

We next consider the case of identical photons at $\Delta \tau = 0$, in this case $\phi (\omega _1, \omega _2) = \phi (\omega _2, \omega _1)$ and $R(\omega _1, \omega _2) = R (\omega _2, \omega _1)$ (because $V_1 = V_2$), and the quantity

12 $$\begin{aligned} P_{1,2}(\Delta \tau =0)=\overline{(T-R)^2} = \int |\phi (\omega _1,\omega _2)|^2 \left( T(\omega _1,\omega _2)-R(\omega _1,\omega _2) \right) ^2 d \omega _1 d \omega _2 . \end{aligned}$$

If in (12) we choose ${\overline{T}} = {\overline{R}} = 1/2$, then we get $P_{1,2} = 4 (\overline{T^2} - {\overline{T}}^2) = 4 (\overline{R^2} - {\overline{R}}^2)$. In other words, there is a mean-square fluctuation of the coefficients of transmission *T* and reflection *R*, which leads to a nonzero value of $P_ {1,2}$ in the case of identical photons. This conclusion is fundamental in the theory of HOM interference and was not previously obtained. Also, from the previously obtained Eqs. (11) and (12) it follows that the $P_{1,2}(\Delta \tau \gg \tau _c)=2\overline{T^2}=2\overline{R^2}$ ($\tau _c$ is the coherence time), as well as $P_{1,2}(\Delta \tau \gg \tau _c)=1/2(1+P_{1,2}(\Delta \tau =0))$.

Let us present the results of calculating the value of $P_{1,2}$ for the case

13 $$\begin{aligned} \phi (\omega _1,\omega _2)=C e^{-\frac{(\omega _1+\omega _2-\Omega _p)^2}{2\sigma ^2_p}}e^{-\frac{(\omega _1-\omega _{01})^2}{2\sigma ^2_1}}e^{-\frac{(\omega _2-\omega _{02})^2}{2\sigma ^2_2}} . \end{aligned}$$

We will be interested in the case applicable for most sources of photons $\omega _{02}-\omega _{01}\ll \omega _{01}, \omega _{02}$; $\omega _{01}/\sigma _1\gg 1$; $\omega _{02}/\sigma _2\gg 1$, in this case the normalization constant $C=\frac{(\sigma ^2_1+\sigma ^2_2+\sigma ^2_p)^{1/4}}{\sqrt{\pi \sigma _1 \sigma _2 \sigma _p}}$. The function (13) allows us to analyze the value of $P_{1,2}$ for two cases that are of practical interest. The first case is spontaneous parametric down-conversion (SPDC), for example, for $\Omega _p = 2 \omega _0; \omega _0 = \omega _{01} = \omega _{02}; \sigma _1 = \sigma _2 = \sigma$ is SPDC of type I, where $\sigma _p$ is the bandwidth of the pump beam, $\omega _0$ and $\sigma$ are the central frequency and the bandwidth, respectively, for both the signal and the idle beams^30^. The second case, on the other hand, if we consider $\sigma _{p}\rightarrow \infty$ in Eq. (13), then this will be the case of Fock states (e.g.^15,30^). Indeed, in this case, in Eq. (13), the $\phi (\omega _1,\omega _2)$ function will be factorized, which corresponds to Fock states. Substituting Eq. (13) into Eq. (11) we obtain

14 $$\begin{aligned} P_{1,2}= \int ^{\infty }_{-\infty }\biggr \lbrace e^{-(y-\frac{\Delta \omega }{\Omega _g})^2} \left( T^2(y)+R^2(y)\right) - 2 B e^{-\left( \frac{\Delta \omega }{\Omega _g}\right) ^2}T(B y)R(-B y) e^{-y^2}\cos \left( B \Delta \tau \Omega _g y \right) \biggr \rbrace \frac{d y}{\sqrt{\pi }}, \end{aligned}$$

where $B=A\sqrt{\frac{1+\frac{\sigma ^2_p}{\sigma ^2_1+\sigma ^2_2}}{A^2+\frac{\sigma ^2_p}{\sigma ^2_1+\sigma ^2_2}}}$ and $B \in (0,1)$; $A=\frac{2\sigma _1\sigma _2}{\sigma ^2_1+\sigma ^2_2};~\Omega _g=\sqrt{\frac{4\sigma ^2_1\sigma ^2_2+(\sigma ^2_1+\sigma ^2_2)\sigma ^2_p}{\sigma ^2_1+\sigma ^2_2+\sigma ^2_p}};~ \Delta \omega =\omega _{02}-\omega _{01}$; *T*(*y*) and *R*(*y*) are determined by the Eq. (8), with the only difference being that

15 $$\begin{aligned} \Omega =\frac{4\pi {\overline{N}} \mathbf{u }_1 \mathbf{u }_2 }{\omega _0\sqrt{V_1 V_2}};~ \epsilon =\frac{\Omega _g}{\Omega }y+\frac{\sqrt{V_2/V_1}-\sqrt{V_1/V_2}}{2 \mathbf{u }_1 \mathbf{u }_2 };~\omega _0=\frac{\omega _{01}+\omega _{02}}{2}. \end{aligned}$$

**Figure 2:**
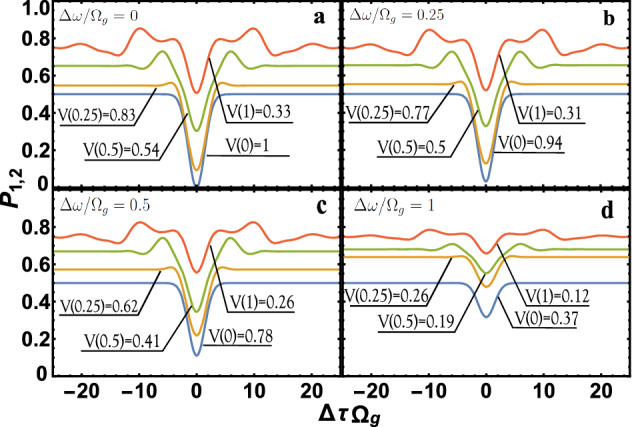
Dependence of $P_{1,2}$ on $\Delta \tau \Omega _g$ (HOM dip). Case **(a)** corresponds to completely identical photons, and cases **(b–d)** correspond to non-identical photons. The visibility $\mathrm{V} = \mathrm{V} (\Omega _g / \Omega )$ depends on the parameter $\Omega _g / \Omega$ (the color of the lines corresponds to: red with $\Omega _g / \Omega =1$, green with $\Omega _g / \Omega =0.5$, brown with $\Omega _g / \Omega =0.25$, blue with $\Omega _g / \Omega =0$). The case $\Omega _g / \Omega = 0$ and visibility $\mathrm{V} (0)$ corresponds to the previously known theory of HOM interference with constant coefficients $T = R = 1/2$.

If we assume that $\Omega _g / \Omega \ll 1$, then *T* and *R* become constant values and they can always be selected in the experiment $T = R = 1/2$. This is true for photon sources where $\Omega _g \ll \Omega$. Estimates of the value of $\Omega$ are given in the conclusion, where it is shown that $\Omega _g$ can be of the order of $\Omega$; therefore, it is necessary to take into account fluctuations of the coefficients R and T. The equation for $P_{1,2}$, in our case Eq. (14) for the constants $T = R = 1/2$ easily integrates and coincides with the well-known $P_{1,2} = 1/2 ( 1-B e^{-(\Delta \omega / \Omega _g)^2} e^{- 1/4 (B \Omega _g \Delta \tau ) ^ 2})$, e.g.^15^. Next, we consider how the value of $P_{1,2}$ will look like depending on $\Delta \tau \Omega _g$ (HOM dip) in the case of $V_1 = V_2, \sigma _1 = \sigma _2$ for different values of $\Omega _g / \Omega$ and $\Delta \omega / \Omega _g$, but for $\Omega t_{BS}$ such that ${\overline{T}} = {\overline{R}} = 1/2$, see Fig. 2: as $\Omega _g / \Omega$ increases, the value of $P_{1,2}$ tends to unity. This can be seen in the general analysis of the Eqs. (8) and (11). Figure 2 also shows that when *T* and *R* are taken into account from the frequency, $P_ {1,2}$ can significantly differ from the previously known theory of HOM interference.

If we assume that photons are completely identical and monochromatic to $\varepsilon =0$ in Eq. (8) (in reality this does not happen), then we get the BS described in^31^ (in this work $R = \sin ^2(C z)$; $P_{1,2} = \cos ^2(2 C z)$; $\phi = \pi /2$, where $C = \Omega /(2v)$ is the coupling constant between adjacent waveguides, $z= v t_{BS}$, *v* is wave velocity in a waveguide). In this case, the frequency dependence of the coefficients *R* and *T* disappears and it is always possible to experimentally select $R = T = 1/2$, where there are no fluctuations of these coefficients. In other words, this case corresponds to the standard HOM theory. You can see that the theory developed here is general, including the one suitable for BS in the form of coupled waveguides. In addition, changing the parameter $\Omega$, you can go to a different type of coupling in the waveguide, including the no coupling waveguide i.e. for $\Omega \rightarrow 0$ we get $T = 1; R = 0$ (photons propagate only along their waveguides). It should be added that such a passage to the limit was not previously in HOM theory (e.g.^31^)

**Figure 3:**
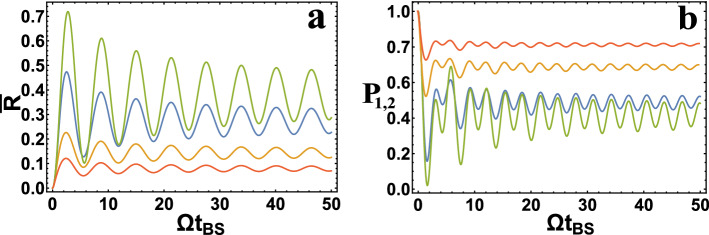
**(a)** The dependence of $$ {\overline{R}} $$ depending on $$ \Omega t_{BS} $$ for $$ \Omega _g / \Omega = 1; 2; 5 ;10$$ (top–down in figure). **(b)** The dependence of $$P_{1,2} $$ depending on $$ \Omega t_{BS} $$ for $$ \Omega _g / \Omega = 1; 2; 5 ;10$$ (bottom–up in figure).

Let us imagine in the figure (see Fig. 3) that in the case of identical photons (but not monochromatic), i.e. for $\sigma _1 = \sigma _2 = \sigma$ (in this case $\Omega _g = \sqrt{2} \sigma$) and $\Delta \omega = 0; \Delta \tau = 0$ the results obtained here may differ from^31^. If in this case we choose $\Omega _g / \Omega \ll 1$, then the dependences *R* and $P_{1,2}$ are simplified and the results coincide with^31^, i.e. $R = \sin ^2 (\Omega t_{BS} / 2)$; $P_{1,2} = \cos ^2 (\Omega t_{BS})$. From Fig. 3 you can see that with the selected parameters, HOM interference ($P_{1,2} \ll 1$) can be realized only with $\Omega _g / \Omega = 1$ and one value $\Omega t_{BS} \approx 2$ . If we take into account that $t_{BS} = z / v$, then we see that HOM interference is possible only for a certain value of length *z*. An estimate will be given below of the value of $\Omega$, where we obtain that *z* should be of the order of micrometers. In the standard HOM theory, there are no restrictions on the length *z* of the waveguide coupling.

It should be added that experiments do not always have good coincidences of $P_{1,2} = P_{1,2}(\Delta \tau )$ with theoretical predictions of HOM interference with constant coefficients $T = R = 1/2$ . In such experimental studies, additional oscillations of the dependence $P_{1,2} = P_{1,2} (\Delta \tau )$ between the minimum of this function and $P_{1,2} = P_{1,2} (\Delta \tau \gg \tau _c)$ ($\tau _c$ is the coherence time). These oscillations can have a different nature (e.g.^32,33^), but fluctuations of the coefficients R and T are not studied. We should also add about the observation in the experiment of the studied effect of fluctuations. If the fluctuations are significant, then the visibility of $\mathrm{V}$ will be small, although the photons can be identical. Therefore, in an experiment without measuring fluctuations they cannot be seen. In other words, if the visibility of the studied source is small, then following the standard HOM theory, we can conclude that the photons are not identical. In fact, this may not be the case, and photons can be identical with large fluctuations. Therefore, the presented theory is needed to avoid such errors in the interpretation of the HOM effect.

## Discussion and conclusion

Thus, the developed theory shows that for a real BS, the coefficients of transmission *T* and the refraction of *R* depend on the frequency. This dependence can significantly change the well-known theory of HOM interference. Under certain conditions, when the dependence on frequencies can be neglected (for example, in the case (Eq. 13) for $\Omega _g / \Omega \ll 1$), the coefficients $T = R = 1/2$ can be selected, and the developed theory is the same as that applying to the case of an ideal BS. In the special case of mixed, identical, and separable photons, there is a relationship between the visibility $\mathrm{V}$ of the HOM dip and the purity ${{\mathscr {P}}}$ of the input photons when $T=R=1/2$^10,17,34–36^

16 $$\begin{aligned} \mathrm{V}=\frac{P_{1,2}(\Delta \tau \gg \tau _c)-P_{1,2}(\Delta \tau =0)}{P_{1,2}(\Delta \tau \gg \tau _c)}=\mathrm{Tr} \rho _1 \rho _2;~~\mathrm{V}( \rho _1= \rho _2)={{\mathscr {P}}}, \end{aligned}$$

where $\rho _1, \rho _2$ are the density matrices of some quantum states of 1 and 2 photons, respectively. If we take into account the dependence of *T* and *R* on frequency, it is easy to see that the dependences of $\mathrm{V} = \mathrm{Tr} \rho _1 \rho _2$ in Eq. (16) will no longer apply (see Eq. (11), where *T* and *R* are present). This leads to the important conclusion that visibility $\mathrm{V}$ with significant fluctuations of the coefficients *T* and *R* cannot be used to judge quantum interference, and for $\rho _1 = \rho _2$ purity ${{\mathscr {P}}}$ of the input photons. Therefore, when conducting an experiment, it is necessary to not only choose ${\overline{T}} = {\overline{R}} = 1/2$, but also minimize fluctuations. It should be added that fluctuations in the HOM interference had not been previously measured, because it was believed that the beam divider had strictly specified coefficients *T* and *R* during the experiment. In the case of HOM interference, the coefficients $T = R = 1/2$ were selected, which actually correspond to ${\overline{T}} = {\overline{R}} = 1/2$ in the experiment. It is quite simple to measure fluctuations at ${\overline{T}} = {\overline{R}} = 1/2$, for this it is necessary to measure $P_{1,2}$ at $\Delta \tau \gg \tau _c$, because, $P_ {1,2} = 2 \overline{T^2} = 2 \overline{R^2}$ (this can be seen from Eq. (11) for $R + T = 1$). If the fluctuations are small, then $P_ {1,2}$ will go over to the known value $P_{1,2} (\Delta \tau \gg \tau _c) = 1/2$ (half of the maximum possible). It is also easy to find visibility $\mathrm{V}$ in the case of identical photons at $T = R = 1/2$ using Eq. (16) and $P_{1,2}(\Delta \tau \gg \tau _c)=2\overline{T^2}=2\overline{R^2}$ , $P_{1,2}(\Delta \tau =0)=4(\overline{T^2}-{\overline{T}}^2)=4(\overline{R^2}-{\overline{R}}^2)$ as $\mathrm{V}_{id}=2{\overline{R}}^2/\overline{R^2}-1=2{\overline{T}}^2/ \overline{T^2}-1$.

A new physical quantity appears in the theory presented, which characterizes BS and its interaction with photons (Eq. 8) is $\Omega$. This value depends on the characteristics of the incident photons: $\omega _1, \omega _2, V_1, V_2, \mathbf{u }_1, \mathbf{u }_2$, and on the characteristics of BS itself ${\overline{N}}$. The frequency $\Omega$ can be estimated if we consider a pair of photons that is quite close in characteristics, i.e. $V_1 \approx V_2 \approx V$, as well as $\omega _1 \approx \omega _2 \approx \omega _0, \mathbf{u } _1 \mathbf{u } _2 \approx 1$ and assume that the overlap of the photon wave packets in BS is ideal (all electrons of atoms with the number ${\overline{N}}$ are in the volume *V*). With such an estimate, it is easy to obtain that $\Omega _{id} = 4 \pi n / \omega _0$, where $n = {\overline{N}}/V$ is the electron concentration in BS ($\Omega _ {id}$ is $\Omega$ in the case of identical photons). It should be added that for of BS, the frequency $\Omega _{id}$ can be represented by the well-known value for plasma frequency $\omega _p$, then $\Omega _{id}=\omega ^2_p/\omega _0$, where $\omega _p=4\pi n e^2/m_{e}$ (in the CGS system). If we quantify $\Omega _ {id}$ for solid materials and the optical frequency ($\sim 10^{15} \mathrm{rad/s}$ ) range, we get that $\Omega _ {id} \sim (10^{14} - 10^{17}) \mathrm{rad/s}$. Of course, the optical frequency is selected as an example, but is not a defining one. The frequency range where fluctuations must be taken into account is much wider. The higher the frequency, the greater the contribution made by the fluctuations of R and T. Obviously, a similar estimate is also valid for non-identical photons; the order in such an estimate will be preserved i.e. $\Omega \sim \Omega _ {id}$. In reality, these $\Omega$ values have lower values due to non-ideal overlap of the wave packets of photons in the BS. It can be seen that these values of $\Omega$ are essential in the theory of HOM interference. For example, from the Eqs. (14) and (15) it can be seen that the dependence of *T* and *R* on frequencies is determined by the relation $\Omega _s / \Omega$, considering the case of optical frequencies of photons with $\omega _0 \sim 10^{15} \mathrm{rad/s}$, where $\Omega _s$ is usually less by orders of magnitude than $\omega _0$ (e.g.^1^, the $\Omega _s \approx 2\pi /\tau _c\approx 10^{14} \mathrm{rad/s}$ value was obtained) we get what could be $\Omega _s / \Omega \sim 1$ (this is the second case with $\omega _2- \omega _1 \sim \Omega$). It can be seen from $\Omega _ {id}\sim 1/\omega _0$ that the developed theory is especially relevant in the case of the ultraviolet and X-ray frequency ranges, since $\Omega _ {id}$ becomes smaller. The frequency $\Omega$ for optical photons can be compared with the spectral line width, for example, for the photon source described in Eq. (13). In other words, compare $\Omega$ with $\sigma _1; \sigma _2$ (for simplicity $\sigma$). Usually, for most photon sources, $\sigma \ll \omega _0$, and as we showed above $\Omega \sim \omega _0$. This means that $\Omega \gg \sigma$, which leads to $\Omega _g / \Omega \ll 1$, where $\Omega _g \sim \sigma$. As mentioned above, if $\Omega _g / \Omega \ll 1$ then our theory coincides with the standard HOM theory with constant coefficients *R*, *T*. This perfectly explains why in the case of BS in the form of prisms, you can usually use the standard HOM theory. In the case when $\Omega \sim \sigma$ this is no longer the case. Such a case can be realized where the coupling $\Omega$ is quite small, for example, on BS in the form of coupled waveguides.

It should be added that the dependence on the frequencies of reflection and transmission coefficients cannot be represented as a function of the optical spectral filters, which are often used in experiments e.g.^37^. Indeed, when using frequency filters, the spectral amplitude (JSA) $\phi (\omega _1, \omega _2)$ is replaced by $\phi ^{'}(\omega _1, \omega _2) = f(\omega _1, \omega _2) \phi (\omega _1, \omega _2)$, where $f (\omega _1, \omega _2)$ is the function optical spectral filters. As can be seen from Eq. (11), the coefficients R and T = 1-R, with respect to $\phi (\omega _1, \omega _2)$ enter non symmetrically, which leads to the above statement. For example, in the case of identical photons, using optical spectral filters, the $P_{1,2}(\Delta \tau =0)=\int |\phi ^{'}(\omega _1,\omega _2)|^2 \left( T-R \right) ^2 d \omega _1 d \omega _2$, for $R = T = 1/2$ we get $P_{1,2}(\Delta \tau =0)=0$. This has fundamental differences from the case of the dependence on the frequencies of the R ant T coefficients, see Eq. (12).
